# “Bumps in the Road”: A Pilot Study of a Therapeutic Technique for the Integration of Unresolved Family Loss and Trauma

**DOI:** 10.3389/fpsyg.2021.635574

**Published:** 2021-06-11

**Authors:** Gabriella J. Watts, Andrew J. Lewis, Irene G. Serfaty

**Affiliations:** Discipline of Psychology, Murdoch University, Murdoch, WA, Australia

**Keywords:** attachment, family therapy, narrative, discourse coherence, defensive processing, segregation

## Abstract

The ability to sustain a coherent narrative about experiences of trauma and loss is a prominent feature of secure-autonomous attachment states of mind as assessed in narrative tasks such as the Adult Attachment Interview. The current study examines the clinical application of the concepts of narrative coherence and discourse segregation within a therapeutic intervention for whole families. *Bumps in the Road* is a family drawing task, which aims to facilitate the co-construction of family narratives about adversities such as trauma, loss and hardship. The technique aims to increase the family’s narrative coherence about such challenging events. The paper first presents a description of the task itself together with the discourse theories of defensive processing of adverse events. The study also presents pilot quantitative findings from 19 parents on the psychometric properties of a coding system of the families’ discourses in undertaking the task and the therapist’s techniques in administering the task. The predictive association of coding of the narratives were examined as predictors of change in internalising and externalising symptoms in the referred child, using the Child Behaviour Checklist. Findings showed that therapist competence in administration of the task did significantly predict the magnitude of treatment efficacy. The current study is the first presentation of this novel therapeutic task and sets a platform for further research on the use of narrative tasks and the formal coding of discourse in therapeutic work with children and families.

## Introduction

The ability to sustain a coherent narrative about challenging interpersonal experiences is a core feature of secure attachment states of mind (Main et al., 2003). In adults, coherence of attachment related discourse can be measured in various ways including attachment-based narratives within structured interviews [Adult Attachment Interview (AAI); Main et al., 1985], projective measures (George and West, 2001), and attachment-based script tasks (Waters and Waters, 2006). In children of verbal age, narrative coherence can be measured using instruments based on story stem tasks (Green et al., 2000). Reviews of the numerous studies of structured interviews such as the AAI suggest that more significant levels of psychopathology are predicted by experiences of past trauma or loss that remain unresolved (Bakermans-Kranenburg and van Ijzendoorn, 2009).

The development of therapeutic techniques to address unresolved attachment discourses could add to the clinical application of such findings. It is a well-established finding that attachment patterns are likely to be transmitted across generations (Verhage et al., 2016). This suggests that the lack of resolution in parent’s discourses on major losses or traumas may influence the way in which families communicate and convey such histories to the next generation. Although many have noted the clinical importance of attachment concepts and findings, the therapeutic application of these principles within well-defined interventions is mostly focused on infant-caregiver dyads (Mountain et al., 2017; Deans, 2020). Despite the importance of the family environment in shaping child mental health outcomes, clinical applications of attachment principles to family interventions are yet to be well established and evaluated as psychological treatment options (Kho, 2020).

The current study attempts to advance such efforts by presenting current work developing a family based therapeutic task called *Bumps in the Road*. This task involves the whole family and is a drawing task that is embedded within a manualised intervention called Behaviour Exchange and Systems Therapy (BEST) (Poole et al., 2017; Lewis, 2020). It is designed to facilitate a discussion that allows a co-construction and integration of family members’ narratives in an effort to address developmental trauma and complicated grief reactions. The task encourages the family to work through their account of challenging family events or circumstances (“bumps”) which may consist of traumas, losses, and major setbacks, in a way that is supported, facilitated and explored by the therapist. The goal is to integrate fragments of memories held by different family members, bringing together possibly incompatible, incomplete or inconsistent accounts of major family events (Lewis, 2020).

### Narrative in Family Systems

The background of the current study is a model of attachment conceptualised as an “attachment-family system,” which reflects an attempt to integrate attachment and family systems theories via the concept of defensive processes occurring in discursive patterns (Lewis, 2020). The attachment-family system operates as a family discourse: a single discursive system which informs each family members’ speech acts as well as how they are interpreted and responded to within that family system (Lewis, 2020). Accordingly, significant incoherence in the family discourse may impede effective communication and connection at points of stress or rupture and thereby perpetuate conflictual or withdrawn interactions. Discursive impasses are likely to be accompanied by incongruent expressions of affect or triggered re-enactments of unresolved traumatic experiences (Lewis, 2020). The coherence of the attachment-family system is conceived as existing on a continuum, with the ultimate goal of the *Bumps in the Road* task being to enhance overall discourse coherence and thereby assist families to resolve challenges using less conflict and greater mutual regulation of affect. Therapy can provide a temporary secure base for the family from which to explore past experiences while actively re-constructing their family narrative to give new meaning to these challenges (Byng-Hall, 1998).

In the current context, “discourse” refers to mostly verbal exchanges which are ordered in a narrative sequence because of the therapist’s initial request to “tell the family’s story.” The family discourse which results is an attempt to generate a “narrative” recounting sequentially the major events in a family’s history. Family narratives can therefore be viewed as a distinct discursive form that functions to retrospectively organise past experiences in the context of dialogues between caregivers and children (Oppenheim, 2006). This process of co-constructing narratives also calls upon the caregiver’s ability to provide emotional scaffolding to support the child’s integration of emotionally laden or complex experiences. Even when children have directly experienced events such as separations, trauma, or loss, lack of resolution within the family discourse can result in the distortion or denial of the necessary information or links between pieces of information to adequately understand events (Oppenheim and Waters, 1995). For example, Bowlby (1988) referenced a number of child therapy cases in which traumatic events were experienced, such as witnessing the suicide of a parent or intrafamilial sexual abuse, and were required by the prevailing family version of events to completely deny their experience. Such conditions may increase the likelihood of holding multiple, contradictory accounts of the same experience (Main, 1993; Lewis, 2020).

### Attachment Representations and Discourse

Attachment theory highlights the influence of early caregiving experiences on one’s psychological and socio-emotional development. Bowlby (1969, 1973, 1980) proposed that early relational experiences with one’s caregiver form a complementary mental representation of the self and others within relationships, that not only informs self-concept, but the prediction and interpretation of future interpersonal interactions, often referred to as an “Internal Working Model” (IWM) (Bowlby, 1973). Main et al. (1985) expanded on this concept and proposed that IWMs are mental representations which comprise cognitive rules for how attachment-related thoughts, feelings and experiences are processed and attended to, with varying degrees of conscious access to such information. This reconceptualisation opened up the possibility of analysing discourse, as spoken by older children and adults, as a means of accessing individual attachment representations.

Main’s insights into the cognitive and linguistic representation of attachment culminated in her development of the AAI, which is based on the speaker’s narrative coherence when describing autobiographical memories around early attachment relationships and experiences of abuse, separation and loss (George et al., 1985). Coherent discourse in an attachment narrative refers to several features, that is, the extent to which discourse is organised and consistent, the speaker is able to easily access and reflect upon attachment experiences, and the collaboration with the interviewer’s interest in their attachment history (Main et al., 2003). The concept of discourse used in Main’s approach was derived from Grice’s (1975, 1989) maxims for cooperative and coherent conversation: quality, quantity, relation, and manner. There is evidence that attachment discourses can be altered by various forms of psychotherapy. Several studies in which the AAI was re-administered at various intervals throughout therapy found that a substantial portion of participants moved from insecure to secure states of mind post-treatment (Fonagy et al., 1996; Stovall-McClough and Cloitre, 2003; Levy et al., 2006; Daniel et al., 2016). Additionally, coherence of discourse and reflective functioning have been found to increase following therapy (Levy et al., 2006). In terms of attachment as a predictor of treatment outcome, research has found that AAI states of mind can differentially predict treatment response as measured by symptom changes. Kuipers et al. (2020) found greater reductions in co-morbid symptoms following therapy in eating disorder patients classified as *Unresolved* pre-treatment. Such findings suggest that symptoms of psychopathology may improve from treatment due to enhanced attachment security, coherence of discourse, and related capacities for affective regulation and reflective functioning (Daniel et al., 2016).

Of particular relevance to the development of *Bumps in the Road* is Main and Hesse’s (1990) striking observations about the discursive manifestations of experiences of trauma or loss in the discourse of adults classified as *Unresolved*. Such discussions may present as contradictory beliefs around key events or experiences of loss, the speaker’s specific role in such events, misattribution of blame, confusion between the speaker’s self-identity and that of the deceased, and disoriented or unintelligible speech with unusual attention to minute detail, or very prolonged silences (Cassidy and Mohr, 2001). Main and Hesse (1990) proposed that such forms of discourse reflect temporary lapses in the speaker’s metacognitive ability to monitor discourse or reasoning, providing glimpses into what they referred to as “non-integrated states of mind.” Such lapses in metacognitive monitoring become more pronounced when challenging content related to attachment cues is introduced (e.g., separations, displays of distress, or themes of loss). An important influence on the development of *Bumps in the Road* was Main and Hesse’s suggestion that such disruptions to discourse coherence impact on the speaker’s capacity to sensitively respond to the current attachment demands of their child. The unresolved parent thereby introduces mismatched or disorienting discourse with their child in relation to the child’s representation of the family’s narrated history of safety, reliability and protection. Our working hypothesis is that such impasses in a shared narrative of family adversity perpetuates implicit meanings that the family is compromised as a secure base or that challenges can be catastrophic or incomprehensible.

Our clinical approach is not simply application of a theory to clinical practice but an attempt to reliably measure features of discourse in family narratives by employing a specific discourse coding modeled on the concept of *defensive exclusion*. This idea was first presented by Bowlby (1980) and is an attempt to synthesise cognitive approaches to information processing with the psychoanalytic concepts of defense proposed originally by Sigmund Freud and elaborated by Anna Freud and Melanie Klein amongst others. *Defensive exclusion* is a process by which incoming sensory information is excluded from consciousness, or retained for a period of time outside of consciousness such that it continues to influence one’s mood, thoughts, or behaviour. It is believed that the function of these processes is to minimise distress when attachment needs are consistently unmet. Bowlby (1980) proposed that such a process could either lead to the attachment system becoming deactivated and attachment-related thoughts and emotions would therefore be diminished or devalued (termed *Deactivation*). Alternatively, the process could lead to affective and/or behavioural responses becoming disconnected from the relational situation eliciting them (termed *Cognitive Disconnection*). *Segregated Systems* was conceived as an extreme defensive process, analogous to splitting, which occurred in response to significant threats to the attachment system. *Segregated Systems* occurs when trauma-related affect and memories are completely blocked from consciousness in order to prevent significant interference with psychological functioning and self-regulation (Bowlby, 1980). Main (1993) proposed that *Segregated Systems* reflect the development of multiple IWMs of the same aspect of reality or experience, however such unintegrated mental models are vulnerable to re-emerging when the attachment system is strongly activated (George and West, 2012). This defense is particularly maladaptive in the long-term due to vulnerability to sudden dysregulation and the emergence of uncontained affect (Juen et al., 2013). Accordingly, *Segregated Systems* have been associated with later psychopathology (Juen et al., 2013) including dissociative responses to traumas (Sroufe, 2005; Liotti, 2006).

The Adult Attachment Projective (AAP) (George and West, 2001) was the first measure to uniquely code narrative discourses for defensive processes in line with Bowlby’s (1980) propositions. All four AAP validation studies examining the four-way classification system undertaken by George and West (2001, 2003, 2012) or their collaborators (Buchheim and George, 2011), found agreement between *Secure-Autonomous*, *Deactivation*, *Disconnection*, and *Segregated Systems* with *Secure-Autonomous*, *Dismissing*, *Preoccupied*, and *Unresolved/Cannot Classify* states of mind on the AAI, with convergence rates ranging from 84 to 94%.

### The Therapist’s Role in Constructing the Family Narrative

Byng-Hall (1995, 1998, 2008) introduced several theoretical concepts which are critical to integrating attachment concepts within a family systems therapy. These include the notions of family scripts, and a “secure family base” as consisting of a network of attachments which at any one time permit all members to feel secure and supported enough for creativity and exploration. Further, Diamond et al.’s (2016) developed an attachment-based family therapy, which integrates principles from several theories and existing modalities including attachment theory and structural family therapy. However, to date, no interventions appear to have focused on the assessment of attachment dynamics within a family system, integrated theories of defensive processing, or attempted to intervene at the level of family discourse structure.

Given the relational focus of attachment-based family therapies and the capacity of the therapeutic relationship to provide a corrective attachment experience, it has been suggested that the therapist may play a valuable role (Slade and Holmes, 2019). Bowlby (1988) proposed that much like a sensitive and responsive attachment figure, the therapist can provide a secure base from which the client can explore painful experiences, provide a sense of safety and validation, offer reflective insights, and enhance the client’s understanding of their internal experience. Within family therapy, the therapist performs the additional function of modelling such capacities to parents so they can, in turn, relate in more adaptive ways to their children (Wright and Edginton, 2016). Research has suggested that aspects of the therapist’s interventions influence therapeutic response. For example, focusing on unmet attachment needs and reframing psychological problems in terms of relational issues supports the processing of vulnerable emotions in adolescents participating in family therapy (Tsvieli et al., 2020), and stronger parent-therapist alliances are associated with increased attachment-promoting behaviour in parents (Feder and Diamond, 2016). These findings highlight the importance of considering therapist variables as a mechanism of change in attachment-based family therapy.

### The Current Study

*Bumps in the Road* is a family based therapeutic drawing task used within an attachment-based family treatment, which aims to facilitate the co-construction and reorganisation of narratives around trauma, loss, and family hardship, with the overarching aim of improving the coherence of the family discourse (Lewis, 2020). This task, unlike the AAP or secure base script tasks (Waters and Waters, 2006) which work largely at the level of implicit memory, aims to integrate implicit (e.g., generalisations about the world) and explicit (e.g., autobiographical) memory, given discrepancies between the two are often met with clinically (Bowlby, 1980). Further, the task is underpinned by theories of defensive processing, particularly segregation, and discourse coherence as clinically meaningful beyond being merely an indicator of attachment security or insecurity.

The current study utilised a mixed-methods approach with an exploratory sequential design to develop a quantitative instrument (Barker et al., 2016). This instrument was intended to code for (1) discursive characteristics of the attachment-family system, specifically, coherence and attachment-related defenses, and (2) characteristics of the therapist’s delivery of the *Bumps in the Road* task as the therapist could have an important role to play in reorganising the family narrative. This quantitative instrument was subsequently applied to video footage of the *Bumps in the Road* task delivered within an six to eight session model, to evaluate scale characteristics, and to establish whether defense patterns in the family discourse and therapist-related variables were associated with treatment responsiveness. Treatment responsiveness was measured by changes in internalising and externalising symptoms in the referred child post-treatment, which was the outcome measure. The latter aim addressed recommendations for more research examining change processes in psychotherapy interventions (Kazdin, 2007).

The hypotheses in the current study were therefore as follows:

(1) As the presence of attachment-related defenses (Deactivation, Disconnection, and Segregation) increased in the family discourse, overall coherence decreased.
(2) The attachment-related defenses as measured by the developed instrument and applied to Bumps in the Road task would correspond with the attachment-related defenses as measured by a similar measure named the Caregiver Attachment Discourse Scale (CADS) which was applied to an attachment-based interview [the Parental Reflective Interview (PRI)] administered in the early sessions of the intervention.
(3) Coding of defense patterns (Coherence, Deactivation, Disconnection, and Segregation) in the family discourse would be associated with change in internalising and externalising symptoms in the referred child post-treatment.
(4) Higher quality intervention delivery by the therapist, would be associated with greater reductions in internalising and externalising symptoms in the referred child post-treatment.

## Materials and Methods

### Part 1: Scale Development

#### Design

**FIGURE 1:**
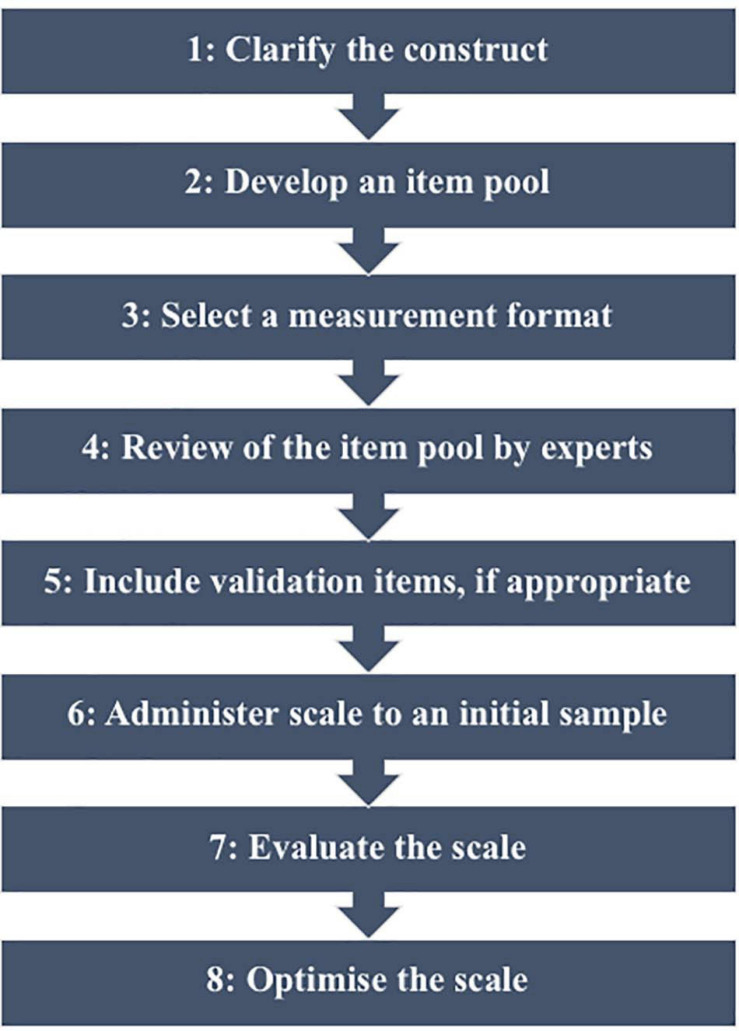
Steps in scale development (DeVellis, 2017).

The current research project was approved by the Murdoch University Human Research Ethics Committee (Project Reference Number: 2020/163). The current study aimed to address Steps 1–6 of DeVellis’ (2017) model for scale development (see Figure 1) in relation to the coding instrument created. Clarification of the construct and development of an item pool were informed by a review of the literature and an initial qualitative analysis and discussion of the videos. The current approach assumed that numerical ratings could be ascribed to characteristics of discourse as an indicator of attachment representations and adequate treatment delivery. However, numerical values were not assumed to be additive and thus the scale of measurement was treated as an ordinal rather than an interval scale (Stevens, 1946). The resulting instrument was reviewed by the research supervisor, AL, who has extensive experience in clinical practice and research in the field of attachment and family therapy. Validation items were not deemed necessary at this point given the scale is clinician-rated, and the administration of additional measures was not possible due to use of pre-collected data. Finally, the scale was applied to the available sample, and initial inter-rater reliability and construct validity were examined. For the purpose of initial scale development, three videos of *Bumps in the Road* were selected from the study data bank, based on perceived variation in family and therapist engagement with the task.

#### Procedure

The analysis of this subset of *Bumps in the Road* videos to develop the scale comprised the qualitative aspect of the current study. The extraction of therapist variables was partially influenced by past research on the role of the therapist in attachment-based family therapies (Slade and Holmes, 2019). The client variables were heavily influenced by the literature and informed by pre-existing codes for the AAI (Main et al., 2003), AAP (George and West, 2001), CADS (Serfaty et al., 2020b), and Manchester Child Attachment Story Task (MCAST) (Green et al., 2000). The subset of videos was analysed using a thematic analysis approach (Braun and Clarke, 2006), which included verbatim transcription, and during which the researcher was blind to outcome data. The resulting quantitative instrument (see Supplementary Material) consisted of the following variables, all scored on seven-point rating scales:

(1) *Therapist: Presentation of the metaphor (Metaphor)* reflected the degree to which the therapist was able to eloquently present *Bumps in the Road* as a metaphor, to convey the task of producing a narrative of the family’s collective experiences. Higher scores reflected greater variation in metaphoric examples provided and the degree to which the therapist conveyed the implied meaning of the task as canvassing and integrating different perspectives of challenging events (1 = Inadequate presentation to 7 = Excellent presentation).
(2) *Therapist: Explanation of the activity and orientation to its therapeutic value (Explanation)* reflected the degree to which the therapist explained the purpose and aims of the activity as discussing and reflecting on the experience of “bumps,” and illustrated how the task is undertaken in a manner that does not bias the family’s responses (1 = Inadequate explanation and orientation to 7 = Excellent explanation and orientation).
(3) *Therapist: Engagement with family discourse (Engagement)* reflected the degree to which the therapist intervened to enhance discourse structure, consequently improving coherence, such as through encouraging elaboration, collaboration, clarification and reflection (1 = Inadequate engagement with discourse to 7 = Excellent engagement with discourse).
(4) *Therapist: Regulation of affect (Affect)* reflected the degree to which the therapist facilitated the family’s expression and understanding of their affective experiences, and provided co-regulation and containment of affect (1 = Inadequate regulation of affect to 7 = Excellent regulation of affect).
(5) *Therapist: Therapeutic space and materials (Therapeutic Space)* reflected the appropriateness of the structure of the task, such as the inclusion of the whole family, the physical environment, and access to materials (1 = Inadequate therapy space and materials to 7 = Excellent therapy space and materials).
(6) *Family Discourse: Coherence (Coherence)* reflected the degree to which the family discourse was consistent, plausible, balanced, and adhered to Grice’s maxims (1975, 1989) for cooperative and collaborative conversation in relation to the aims of the task (1 = Not coherent to 7 = Highly coherent).
(7) *Family Discourse: Deactivation (Deactivation)* reflected the degree to which defensive deactivation appeared evident in the family discourse, such as through tendencies to minimise the presence and severity of difficult experiences, or the dismissal of another’s attachment needs (1 = No evidence of deactivation to 7 = Definite evidence of deactivation).
(8) *Family Discourse: Disconnection (Disconnection)* reflected the degree to which defensive disconnection appeared evident in the family discourse, such as through heightened emotion or excessive elaboration (1 = No evidence of disconnection to 7 = Definite evidence of disconnection).
(9) *Family Discourse: Segregation with respect to Trauma (Segregation [Trauma])* and *Segregation with respect to Loss (Segregation [Loss])* reflected the degree to which defensive segregation appeared evident in the family discourse during specific discussions of trauma and loss, respectively, such as through emotional or behavioural dysregulation or constriction, or odd and disorganised discourse (1 = No evidence of segregation to 7 = Definite evidence of segregation).

### Part 2: Scale Evaluation and Application

#### Participants

The total sample comprised 14 children (52.6% males and 47.4% females) aged 6 to 12 (*M* = 9, *SD* = 2.08) and their families, who completed *Bumps in the Road* as part of BEST interventions, specifically BEST-Foundations (BEST-F; Benstead and Lewis, 2016) (*N* = 6) or the Foundations Intervention (Serfaty et al., 2020a) (*N* = 8). For five of these families, CADS data was obtained from both caregivers (therefore *N* = 19 in the relevant analyses). Children were recruited from referrals to the Murdoch Psychology Clinic and were eligible on the basis that the child was presenting with at least sub-clinical levels of depression and/or anxiety as measured on the Child Behaviour Checklist (CBCL) and met the age requirements for the respective study. Exclusion criteria included children presenting with other specific mental health issues or neurodevelopmental disorders, caregivers presenting with serious mental health issues impeding their ability to participate in the intervention, and families unable to adhere to participation requirements.

#### Measures

##### Child behaviour checklist (CBCL) (Achenbach and Rescorla, 2001)

The CBCL is a parent-report measure of emotional and behavioural problems in children aged between 6 and 18 years. The CBCL consists of 113 items, measured on a three-point Likert scale (0 = Not True, 1 = Somewhat or Sometimes True, 2 = Very True or Often True), and provides norm-referenced scores in relation to a number of scales, including Internalising and Externalising scales. The psychometric properties of the CBCL reflect high internal consistency and test–retest reliability for the Internalising (*a* = 0.90, *r* = 0.91) and Externalising (*a* = 0.94, *r* = 0.92) scales (Achenbach and Rescorla, 2001). Additionally, construct validity of the measure is supported through findings of high correlations (ranging from 0.80 to 0.88) between the Internalising and Externalising scales on the CBCL and the Behaviour Assessment System for Children (BASC) (Reynolds and Kamphaus, 2002), a brief screening measure of emotional and behavioural problems in children.

##### Caregiver attachment discourse scale (CADS) (Serfaty et al., 2020b)

The CADS is a newly developed discourse-based coding measure for application to a structured parent interview. While the CADS is similar to the coding tool developed in the current study, it differs in that the coding is applied on an individual caregiver rather than a whole family and encapsulates only the attachment-related defenses, *Deactivation*, *Disconnection*, and *Segregation*, as they present in the caregiver’s discourse. The presence of these three defenses in discourse is scored on a three-point scale (0 = No concern, 1 = Possible Concern, and 2 = Definite Concern), in relation to the caregiver’s attachment relationships with their mother of origin, father of origin, partner, and child. For the purposes of the current study, CADS scores for each caregiver comprised the total number of attachment relationships in which a particular defense was present to a level of *Definite Concern*. Therefore, scores ranged from 0 to 4. Initial evaluation of the psychometric properties of the CADS have indicated moderate to excellent inter-rater reliability (Koo and Li, 2016), in relation to coding for the presence of *Deactivation* (ICC = 0.75, 95% CI = 0.46–0.92), *Disconnection* (ICC = 0.93, 95% CI = 0.82–0.98), and *Segregation* (ICC = 0.86, 95% CI = 0.64–0.96) in the caregiver’s discourse to a level of definite clinical concern (Serfaty et al., 2020b).

#### Procedure

##### Administration of outcome measure

In BEST-F, the CBCL was administered at baseline (4 weeks prior to the commencement of treatment), pre-treatment (session one), post-treatment (session eight), and at follow-up (8 weeks following treatment). In the Foundations Intervention, the CBCL was administered at baseline (2 weeks prior to commencement of treatment), pre-treatment (session one), mid-treatment (session three), post-treatment (session six), and at follow-up (4 weeks following treatment). For the purposes of the current study, the “change score” reflected follow-up CBCL scores minus baseline CBCL scores.

##### Interventions

BEST-F and the Foundations Intervention were implemented by Masters and Doctorate Clinical Psychology students between 2016 and 2020 as part of research trials, at the Murdoch Psychology Clinic in Western Australia. BEST-F is an 8-session manualised intervention which adopts techniques from previous BEST interventions and applies them to a whole family therapy approach. The treatment emphasises the significance of the family’s social environment in perpetuating the child’s mental health difficulties, and therefore aims to improve family communication, the quality of interactions, reflective functioning, emotion regulation, and parental self-care. The Foundations Intervention consists of six sessions and is designed to target emotional and behavioural problems in children aged 6–12 years, recognising the paucity of well-established attachment-based interventions targeting this population. *Bumps in the Road* was conducted at Session 5 of BEST-F and Sessions 5 or 6 of the Foundations Intervention. All sessions were videotaped for quality and research purposes.

##### “Bumps in the road” session

*Bumps in the Road* is a drawing activity undertaken as a whole family task which uses the metaphor of a car driving along a “rocky” road where there are “bumps,” “pot-holes” and various accidents along the way, and people who “jump on board” or “jump out” of the car (Lewis, 2020). A simple metaphor was deliberately selected for the task to enable externalisation and distancing from problems, and to facilitate discussion of painful experiences in a playful and contained way which is likely to be more appealing to children (Legowski and Brownlee, 2001). Family members were encouraged to label the “bumps” encountered along their journey, which represent any challenges, traumas, or significant losses experienced by the family. The metaphor should extend to capture the varying consequences that may occur when the car encounters a “bump,” including that it may break down and require repairs, or that family members may fall out of the car for periods of time or indefinitely. The activity provides a medium through which collaborative discussion can occur around difficult events, and family members can better understand the impact of these events on each person, enhancing reflective capacity. Further, the activity provides opportunity for the therapist to actively prompt for elaboration, clarify inconsistencies, and encourage reflection, with the overarching aim of improving the coherence of the family’s narrative around difficult events.

##### Inter-rater agreement

The process of establishing the degree of inter-rater agreement for the developed scale, was predominantly informed by Adler et al. (2017)’s article on narrative research. Two post-graduate clinical psychology students, both of whom were familiar with BEST either through training and/or delivery of the intervention, participated as comparative raters. Raters firstly participated in a 2-h training around the development, rationale, and application of the coding scale. The raters were subsequently required to code an initial video independently which was excluded from the data set, and any discrepancies or queries were addressed in a second 2-h training session. Coders then independently coded three videos which were chosen at random. A third 2-h training session occurred in which significant discrepancies within the coding for these three videos were discussed, consensus was reached, and minor refinements to the wording of the coding scale were made; these three videos were included in the final dataset. Finally, the comparative raters individually coded a subset of the videos (one rater coded an additional two videos and the second rater coded an additional seven videos). The raters were blind to the original coder’s ratings and were provided with the family’s genogram and some basic background information (such as the presenting problem, and any significant events the family had disclosed in prior sessions). The resulting coding was compared to that of the original coder and developer of the scale. Inter-rater reliability was evaluated through the percentage of ratings ascribed by comparative coders within one point of the original coder, and whereby agreement of above 70% (“fair” and above) was deemed acceptable for the purposes of this pilot study (Cicchetti, 2001). Additionally, intra-class correlations (ICC) were interpreted in line with McGraw and Wong (1996), with values over 0.5 considered acceptable for the purposes of the current study.

#### Data Analysis Approach

##### Data extraction and screening

The quantitative instrument developed in Part 1 of the current project was applied to the full available sample to extract quantitative data for each of the nine variables. Data was found to be missing in relation to follow-up CBCL data from three caregivers, in which cases post-intervention CBCL scores were utilised rather than imputing the missing data points with predicted values. Additionally, all CBCL data was missing in relation to another participant; this participant was therefore excluded from analyses which utilised CBCL scores. Univariate outliers were analysed through visual inspection of the boxplots, and computing standardised scores for the two continuous outcome variables – changes in Internalising and Externalising CBCL scores. No univariate outliers were indicated by visual inspection of box plots or on the basis of *z* scores above or below 3.29 *SD*s (*p* < 0.001) from the mean (Tabachnick and Fidell, 2007). Visual inspection of the scatter plots did not indicate the presence of non-linear relationships between the variables. Finally, skew and kurtosis statistics were observed for each variable to explore the spread of scores, with scores considered to vary significantly from normality based on the ratio of skew or kurtosis to standard error ≥±3.29 (Tabachnick and Fidell, 2007).

##### Data analysis and interpretation

Statistical analyses were conducted using IBM SPSS statistics software. In order to test the hypothesised relationships between the variables, bivariate Spearman’s correlations were analysed due to the ordinal nature of the rating scales. In an effort to reduce the likelihood of Type II errors resulting from the small sample size, exact *p* values were reported and moderate to large correlations, regardless of statistical significance, were examined and discussed. Accordingly, data was considered to support the hypothesis if statistical significance at an alpha level of *p* < 0.05 was indicated, or if Spearman’s correlations were equal or greater than 0.3, in line with Cohen’s (1992) criteria for moderate effect sizes. Further, to enhance interpretability, scatterplots were examined where data was found to support the hypothesis. Lastly, both quantitative data and qualitative observations were integrated in the discussion to provide a more comprehensive understanding of the task.

## Results

### Descriptive Statistics

Table 1 displays the mean scores, median scores, standard deviations, minimum and maximum scores, and skew and kurtosis statistics for nine variables derived from the coding instrument, and the CBCL Internalising and Externalising change scores. Of note, the positively skewed distribution of the Segregation (Loss) variable may prove problematic as scores appear to significantly deviate from normality. This observation may be due to the small sample size and may limit the statistical power of the analyses due to limited variability of the scores.

**TABLE 1:** Descriptive statistics for the therapist variables (Metaphor, Explanation, Engagement, Affect, and Therapeutic Space), family discourse variables [Coherence, Deactivation, Disconnection, Segregation (Trauma), and Segregation (Loss)], and the change scores (CBCL Internalising and CBCL Externalising).

	Mean	Std. Error of Mean	Median	Standard Deviation	Minimum	Maximum	Skew	Std. Error of Skew	Kurtosis	Std. Error of Kurtosis
Metaphor	4.74	0.25	5.00	1.10	3	6	–0.54	0.52	–0.91	1.01
Explanation	4.05	0.26	4.00	1.13	2	6	–0.63	0.52	–0.05	1.01
Engagement	3.21	0.26	3.00	1.13	2	6	1.07	0.52	0.77	1.01
Affect	3.58	0.31	3.00	1.35	2	7	1.19	0.52	1.13	1.01
Therapeutic Space	4.32	0.37	4.00	1.60	2	7	0.15	0.52	–0.92	1.01
Coherence	2.89	0.26	3.00	1.15	1	6	0.72	0.52	2.02	1.01
Deactivation	4.42	0.40	4.00	1.74	1	7	–0.17	0.52	–0.54	1.01
Disconnection	2.63	0.38	2.00	1.67	1	6	0.42	0.52	–1.22	1.01
Segregation (Trauma)	2.26	0.43	1.00	1.88	1	6	1.09	0.52	–0.45	1.01
Segregation (Loss)	2.11	0.40	1.00	1.76	1	7	1.87	0.52	3.08	1.01
CBCL Internalising^*a*^	–13.11	1.73	−13	7.35	−2	−27	–0.20	0.54	–0.76	1.04
CBCL Externalising^*a*^	–9.06	1.25	−8.5	5.31	0	−17	0.05	0.54	–0.96	1.04

*N = 19. ^*a*^*N* = 18.*

### Inter-Rater Reliability

Inter-rater reliability was evaluated in relation to the 15 pairs of observations which were available. The percentage of agreement between the coders, differences between the mean values of coding for each coder, and the intra-class correlation coefficients are displayed in Table 2. The percentage of agreement between coders was observed to be excellent for the Metaphor variable, good for the Explanation, Affect, Therapeutic Space, Coherence, Deactivation, and Segregated Systems (Loss) variables, fair for the Segregated Systems (Trauma) variable, and poor for the Engagement and Disconnection variables (Cicchetti, 2001). The differences in mean score between the coders indicates that the comparative coder provided very similar, but overall higher scores on all variables (with the exception of Segregation), relative to the original coder. The ICC indicated good reliability in relation to the Coherence variable, moderate reliability in relation to the Metaphor, Affect, Disconnection, and Segregated Systems (Trauma) variables, and poor reliability in relation to the Explanation, Engagement, Therapeutic Space, Deactivation, and Segregated Systems (Loss) variables (McGraw and Wong, 1996).

**TABLE 2:** Inter-rater reliability statistics: percentage agreement (% Agreement), mean scores for the original coder (M1) and comparative coder (M2), the difference between these two mean scores (diffM), and the intra-class correlation (ICC) coefficient.

	Metaphor	Explanation	Engagement	Affect	Therapeutic Space	Coherence	Deactivation	Disconnection	Segregated Systems (Trauma)	Segregated Systems (Loss)
% Agreement	93	80	67	80	80	87	80	67	73	93
M1	4.47	3.67	3.33	3.67	4.07	2.87	4.20	3.13	2.67	1.47
M2	4.80	3.87	4.2	4.47	4.40	2.93	5.00	3.20	1.87	1
diffM	−0.33	−0.20	−0.87	−0.80	−0.33	−0.07	−0.80	−0.07	0.80	0.47
ICC	0.75	0.05	0.04	0.51	0.32	0.81	0.49	0.65	0.65	−0.01

*N = 15 (total comparisons).*

### Hypothesis One: Family Discourse Coherence and Attachment Defenses

**FIGURE 2:**
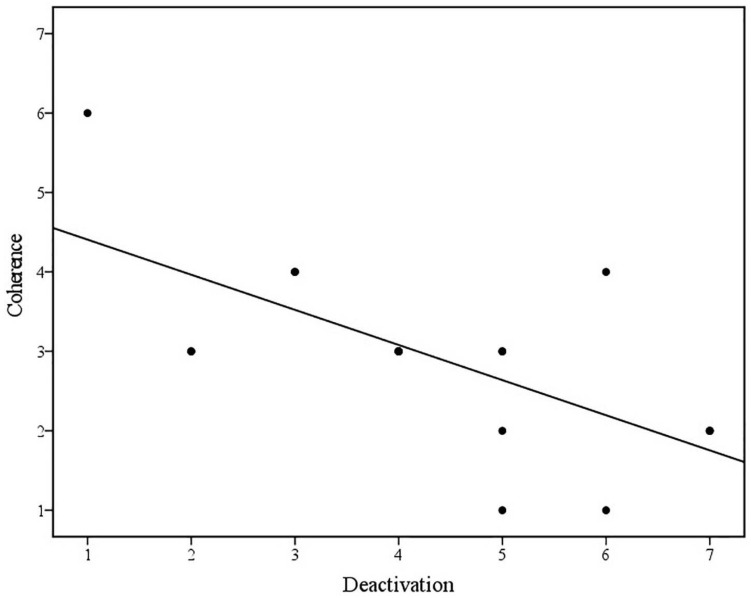
Scatterplot and line of best fit (linear) illustrating the association between Coherence of family discourse and Deactivation (*N* = 19) (Linear equation: *y* = 4.85–0.44×; *R*^2^ = 0.45).

**FIGURE 3:**
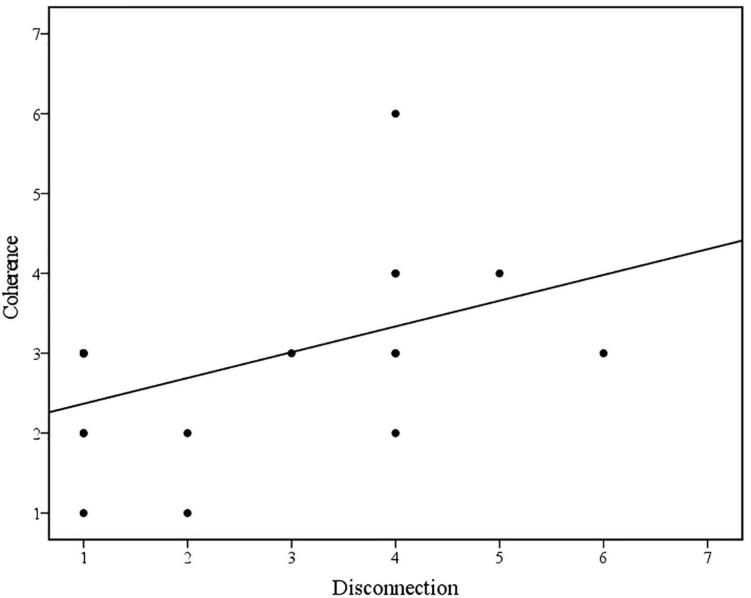
Scatterplot and line of best fit (linear) illustrating the association between Coherence of family discourse and Disconnection (*N* = 19) (Linear equation: *y* = 2.05+0.32×; *R*^2^ = 0.22).

Bivariate Spearman’s correlations between Coherence and defenses in the family discourse are displayed in Table 3. Spearman’s rho indicated a statistically significant negative correlation between Coherence and Deactivation. Visual inspection of the scatter plot and line of best fit (displayed in Figure 2) indicated that as the presence and severity of defensive deactivation increases, the overall coherence of the family discourse decreases. This was found to be a large effect size (Cohen, 1992) and partially supports hypothesis one. However, contrary to hypothesis one, Spearman’s rho indicated a statistically significant positive correlation between Coherence and Disconnection. Visual inspection of the scatter plot and line of best fit (displayed in Figure 3) indicated that as the presence and severity of disconnection increases, the overall coherence of the family discourse increases. This was found to be a large effect size by Cohen’s (1992) criteria.

**TABLE 3:** Spearman’s correlations (r_*s*_) of coherence of the family discourse with other attachment-related defenses.

	1	2	3	4	5
1. Coherence		−0.67** *p* = 0.002	0.48* *p* = 0.04	−0.19 *p* = 0.431	−0.20 *p* = 0.425
2. Deactivation			−0.33 *p* = 0.168	0.18 *p* = 0.468	0.35 *p* = 0.144
3. Disconnection				0.12 *p* = 0.517	−0.25 *p* = 0.294
4. Segregation (Trauma)					−0.007 *p* = 0.767
5. Segregation (Loss)					

N = 19; *p < 0.05, **p < 0.001.

Small and non-significant associations were found between Coherence and Segregation (Trauma), and Coherence and Segregation (Loss), however the direction of the association was negative, consistent with the first hypothesis.

### Hypothesis Two: Attachment Defenses Evident in Bumps in the Road, and as Measured by the CADS

**FIGURE 4:**
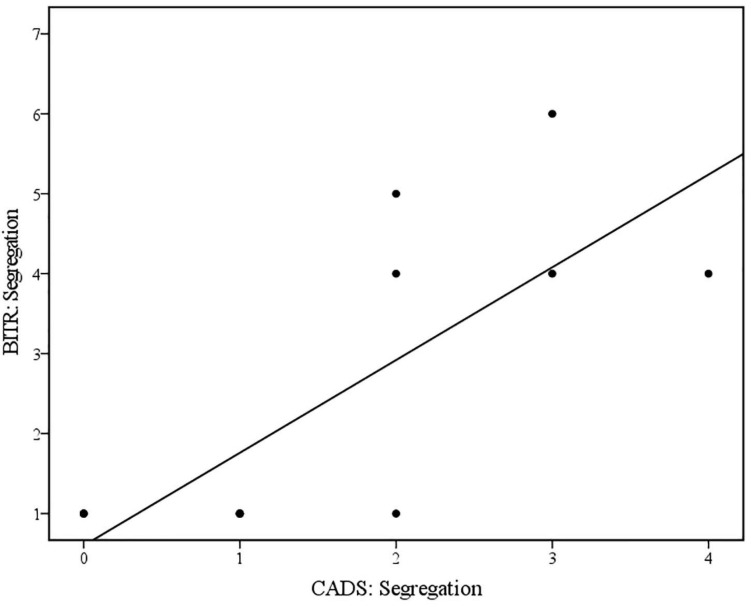
Scatterplot and line of best fit (linear) illustrating the relationship between Segregation (Trauma) in Bumps in the Road (BITR: Segregation), and Segregation as measured by CADS (CADS: Segregation) (*N* = 13) (Linear equation: *y* = 0.6+1.16×; *R*^2^ = 0.60).

Bivariate Spearman’s correlations between coding for defenses derived from the developed coding instrument and from the CADS are displayed in Table 4. Spearman’s rho indicated a statistically significant positive correlation between Segregation (Trauma) as evident in *Bumps in the Road*, and Segregation as measured by the CADS. Visual inspection of the scatter plot and line of best fit (displayed in Figure 4) indicated that higher scores for Segregation in relation to trauma in *Bumps in the Road* was associated with higher Segregation scores on the CADS. This was found to be a large effect size (Cohen, 1992) and partially supports the second hypothesis. No other significant relationships were observed between coding for attachment-related defenses in *Bumps in the Road* and on the CADS.

**TABLE 4:** Spearman’s correlations (r_*s*_) of attachment−related defenses in Bumps in the Road (BITR) with attachment−related defenses as measured by the CADS.

	CADS: Deactivation	CADS: Disconnection	CADS: Segregation
**BITR: Deactivation**	0.24 *p* = 0.425	−0.03 *p* = 0.921	0.00 *p* = 0.996
**BITR: Disconnection**	−0.26 *p* = 0.395	0.11 *p* = 0.73	0.36 *p* = 0.229
**BITR: Segregation (Trauma)**	−0.22 *p* = 0.471	0.48 *p* = 0.097	0.80** *p* = 0.001
**BITR: Segregation (Loss)**	0.54 *p* = 0.055	−0.46 *p* = 0.118	−0.12 *p* = 0.688

N = 13; **p < 0.001.

### Hypothesis Three: Defense Patterns in Family Discourse as Predictors of Change in Child Symptoms

Bivariate Spearman’s correlations between Coherence, Deactivation, Disconnection, Segregation (Trauma), and Segregation (Loss), and changes in internalising and externalising CBCL scores across treatment are displayed in Table 5. Contrary to the hypothesis, none of the defense processes in the family discourse during *Bumps in the Road* appeared to be associated with change in internalising or externalising symptoms in the referred child post-treatment (*r*_*s*_ > 0.3 and/or *p* < 0.05).

**TABLE 5:** Spearman’s correlations (r_*s*_) of defense processes [Coherence, Deactivation, Disconnection, Segregation (Trauma), and Segregation (Loss)] and the CBCL change scores (Internalising and Externalising).

	Change in Internalising Symptoms	Change in Externalising Symptoms
Coherence	0.06; *p* = 0.816	0.07; *p* = 0.775
Deactivation	0.20; *p* = 0.439	−0.04; *p* = 0.888
Disconnection	−0.19; *p* = 0.459	0.18; *p* = 0.481
Segregation (Trauma)	−0.19; *p* = 0.442	0.09; *p* = 0.712
Segregation (Loss)	−0.04; *p* = 0.878	−0.24; *p* = 0.338

*N = 18.*

### Hypothesis Four: Therapist Variables as Predictors of Change in Child Symptoms

**FIGURE 5:**
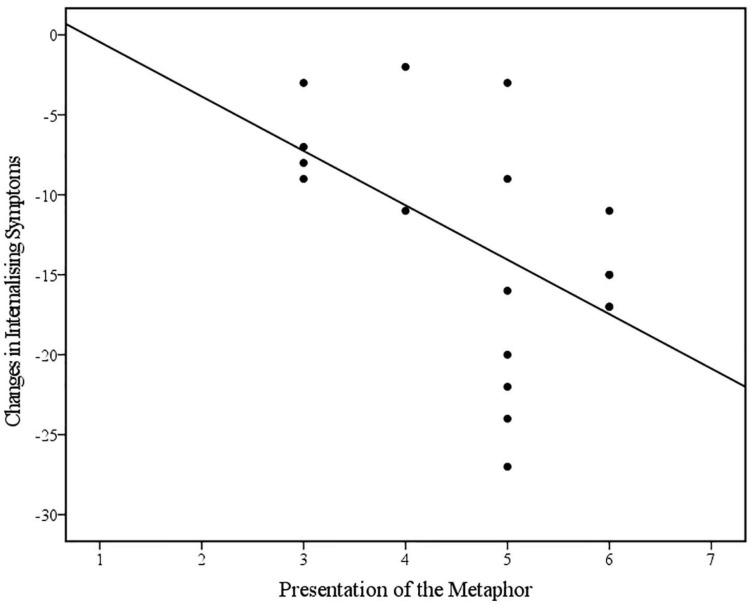
Scatterplot and line of best fit (linear) illustrating the relationship between the therapist’s Presentation of the Metaphor and changes in Internalising Symptoms on the CBCL across treatment (*N* = 18) (Linear equation: *y* = 2.96–3.4×; *R*^2^ = 0.27).

**FIGURE 6:**
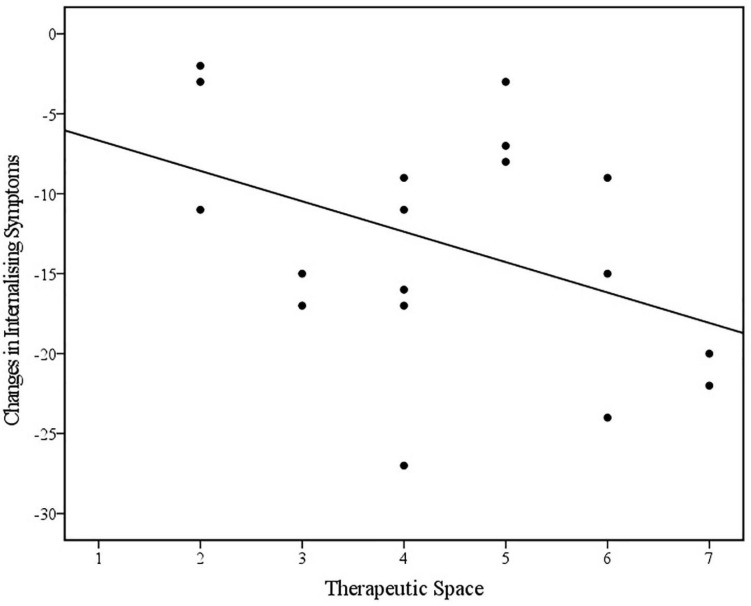
Scatterplot and line of best fit (linear) illustrating the relationship between use of Therapeutic Space and changes in Internalising Symptoms on the CBCL across treatment (*N* = 18) (Linear equation: *y* = –4.76–1.9×; *R*^2^ = 0.18).

Bivariate Spearman’s correlations between therapist variables (Metaphor, Explanation, Engagement, Affect, and Space) and changes in internalising and externalising CBCL scores across treatment are displayed in Table 6. Spearman’s rho indicated a statistically significant positive correlation between the therapist’s use of metaphor in *Bumps in the Road* and change in internalising symptoms in the referred child post-treatment. Visual inspection of the scatter plot and line of best fit (displayed in Figure 5) indicated that greater reductions in internalising symptoms were associated with how well the therapist presented the metaphor. This was found to be a large effect size (Cohen, 1992) and partially supports the hypothesis. Spearman’s rho additionally indicated a negative correlation, albeit non-significant, between the therapeutic space and materials used during *Bumps in the Road* and change in externalising symptoms in the referred child post-treatment. Visual inspection of the scatterplot and line of best fit (as displayed in Figure 6) indicated that better structure of the therapeutic space and availability of materials, was associated with greater reductions in externalising symptoms. This was found to be a medium effect size (Cohen, 1992) and partially supports the hypothesis. Contrary to the hypothesis, no other therapist variables were found to be associated with change in internalising or externalising symptoms in the referred child post-treatment (*r*_*s*_ > 0.3 and/or *p* < 0.05).

**TABLE 6:** Spearman’s correlations (r_*s*_) of therapist variables (Metaphor, Explanation, Engagement, Affect, and Therapeutic Space) and the CBCL change scores (Internalising and Externalising).

	Change in Internalising Symptoms	Change in Externalising Symptoms
Metaphor	−0.54*; *p* = 0.022	−0.13; *p* = 0.601
Explanation	−0.12; *p* = 0.639	0.26; *p* = 0.293
Engagement	−0.13; *p* = 0.595	0.24; *p* = 0.336
Affect	−0.01; *p* = 0.967	0.22; *p* = 0.375
Therapeutic Space	−0.35; *p* = 0.155	−0.29; *p* = 0.238

N = 18; *p < 0.05.

## Discussion

The current study was a pilot and it aims to describe a therapeutic task, *Bumps in the Road*, present the initial stages of the development a family based coding instrument for attachment discourse at a family systems level and apply this instrument to the *Bumps in the Road* task. The paper aimed to explore which aspects of the family discourse and therapist’s interventions were associated with treatment outcomes for children. An exploratory sequential mixed-methods design was adopted to develop the coding instrument, initial inter-rater reliability was established, and Spearman’s correlations were explored in order to evaluate hypothesised relationships between the developed variables both internally and externally. The findings and implications relating to each hypothesis are discussed below.

### Inter-Rater Reliability

Overall, the inter-rater reliability analyses indicated adequate support for several variables measured by the coding scale and some variables in need of further development. The therapist variables of Metaphor and Affect, and the attachment defense variables of Coherence and Segregated Systems (Trauma) performed reasonably well. The interpretability of inter-rater reliability statistics in relation to the remaining variables was complicated by notable differences between the percentage agreement and intra-class correlation coefficients, which was likely impacted by range restriction and small sample size.

Further, findings of suboptimal agreement within some of the variables highlighted areas for further refinement of the scale. For example, throughout the process of establishing inter-rater agreement, coding for the therapist’s engagement with discourse appeared to be more prone to subjective interpretations, contributing to inconsistency between raters. This variable relied on the coder using their clinical judgment to determine the degree to which the therapist’s engagement with the family discourse was hindered by the family’s response (thus rating the therapist’s performance as higher), or to what degree the therapist should have persisted with attempts to work therapeutically with the discourse unfolding throughout the activity (thus rating the therapist’s performance lower). Likewise, coding for the Disconnection variable was met with a level of disagreement around the degree to which the therapist exacerbated certain discourse characteristics, such as perpetuating over-elaborative responses by further prompting around irrelevant details of events. Coders were therefore encouraged to partition out the therapist’s involvement as much as possible, in order to code for family discourse independent of these factors. This issue may also be addressed in future through further clarifying the aims specific to the *Bumps in the Road task* and providing clear guidance to the therapist (in other words, further standardising the administration of the task).

Another interesting observation which arose through the process of establishing inter-rater agreement, was the difficulty in coding defensive processes at a family level. While there was a good degree of consensus regarding coding for coherence of family discourse, the agreement between coders for indicators of defensive discourse was poor. It appeared that coders were able to agree that the family’s discourse and interactions were incoherent, but the specific forms of incoherence were more difficult to discern, particularly with family members often presenting with different individual defenses. This observation may speak to the view that attachment-related defenses only operate on an individualised level, rather than an intersubjective or interpersonal one. Further research is therefore needed to explore how attachment can be conceptualised and operationalised at the level of the family system as a whole.

### Hypothesis One: Family Discourse Coherence and Attachment-Related Defenses

The first hypothesis was partially supported, and it was found that as deactivation increased in the family discourse, coherence decreased. This was a large, statistically significant association. While no other relationships were found between segregation and coherence, the negative direction of the relationship supported the hypothesis. Interestingly, results also indicated that as disconnection increased in the family discourse, so did coherence; this was similarly a large, statistically significant association. This finding is contrary to the hypothesis and points to the need for further refinement of the Disconnection variable, particularly due to results indicating poor inter-rater agreement around coding for this variable at the family system level. Due to the content and structure of *Bumps in the Road* discourse differing markedly from previous discourse-based assessments, indicators for all defensive processes were adapted based on how such processes were theorised to present in the context of the task. The variation of scores for Disconnection from the original coder, indicated that only approximately 10% of participants scored above the mid-point of “Possible evidence of Disconnection.” This highlights a lack of clarity in how Disconnection may present in the narratives of whole families, such that discourse with possible evidence of defensive disconnection may still have been scored highly for coherence. This finding also highlights a need to revise the procedure by which the coding instrument is applied, such that coding for coherence should occur after, and taking into account, coding for attachment-related defenses.

### Hypothesis Two: Attachment-Related Defenses Evident in Bumps in the Road, and as Measured by the CADS

For the second hypothesis, it was found that Segregation in relation to trauma in *Bumps in the Road*, was significantly correlated with Segregation as measured by the CADS; this was a large, statistically significant association. There are several factors which may account for the lack of concordance between the two rating scales. Firstly, the CADS coding reflected one caregiver’s discourse as opposed to the family discourse in *Bumps in the Road*. Secondly, the structure of the task itself limited opportunities to observe defensive processes relative to semi-structured attachment interviews which are more explicit in eliciting discursive styles; and finally, families received 3–4 sessions of active therapy between PRI administration and the *Bumps in the Road* task which may have led to changes in discourse structure and defensive relating. Despite this, findings of a large association between Segregation as measured by both rating scales may highlight the pervasiveness of this particular defense within the attachment-family system, such that for individual caregivers presenting with features of unresolved trauma in attachment-based interviews, that unresolved trauma appears to be similarly permeating the family discourse. How the presence of segregated family discourse may give rise to disorganisation in the child is beyond the scope of this analysis however, this finding supports the working hypothesis stated above that attachment disorganisation may be held in place by and transmitted by specific features of the family’s discourse (Lewis, 2020). It also infers that the presence of unresolved trauma in at least one caregiver may be a significant risk factor for disorganisation in the child regardless of whether it is present in the individual discourse of the other caregiver. It is however acknowledged that there were few cases in which unresolved trauma was only present in the discourse of fathers during the PRI, and this finding may therefore reflect the stronger influence of maternal compared to paternal attachment representations on those of their children (Bretherton, 2010).

### Hypothesis Three: Coding of Defense Patterns in Family Discourse and Change in Internalising and Externalising Symptoms in the Referred Child

In relation to the third hypothesis, there were no findings of meaningful or statistically significant associations between defensive patterns in the family discourse and changes in internalising or externalising symptoms in the referred child post-treatment. This suggests that discursive features of particular attachment-related defenses within the attachment-family system during *Bumps in the Road* were not associated with changes in the referred child’s mental health as a result of treatment. In this small dataset, coherence of discourse and defensive patterns during the task were not predictive of treatment response.

This finding contradicts past research which has found coherence and attachment classifications to predict treatment outcome (Fonagy et al., 1996; Levy et al., 2006; Daniel et al., 2016; Kuipers et al., 2020). It may be the case that change processes within the attachment-family system from therapy are multifaceted and more complex than what can be gleaned from research on individual psychotherapy. Furthermore, two major methodological limitations may explain the lack of support for this hypothesis. Firstly, the small sample size of the current study limited the power of the analysis to detect even moderate associations between the variables. Secondly, where possible, CBCL data was collected from both caregivers in relation to the same child and in the majority of these cases, CBCL data was observed to differ, sometimes significantly, between caregivers. Discrepancies between mother and father CBCL ratings are similarly reflected in the literature, with mothers often reporting higher levels of symptomatology (Schroeder et al., 2010), and may be due to differences in mother’s and father’s styles of attribution, implicit definitions of child “problems,” or differences in their degree of exposure to problem behaviours. Given that differing CBCL scores were assigned to the same coding for family discourse, any clear associations between defensive patterns and CBCL change scores may therefore be less discernible. While assessing child emotional and behavioural problems from multiple informants offers valuable insights, a larger sample would have permitted use of multi-level modelling to address this limitation.

### Hypothesis Four: Therapist Variables and Change in Internalising and Externalising Symptoms in the Referred Child

In relation to the fourth hypothesis, results indicated that higher therapist competence in presenting the *Bumps in the Road* metaphor was associated with greater changes in internalising symptoms in the referred child; this was a large, statistically significant association. Additionally, it was found that better structure of the therapeutic space was associated with greater changes in internalising symptoms in the referred child, although this was a small, non-significant association. The former finding would be consistent with a view that therapist competence in administering *Bumps in the Road* is an important factor relating to treatment responsiveness. This finding can be interpreted as an indication that the presentation of the “bumps” metaphor may be fundamental to the effectiveness of the task. However, it is also acknowledged that therapists may have displayed a similarly high level of competence delivering other aspects of the intervention. The latter finding that therapeutic space is associated with treatment outcome aligns with past research highlighting the importance of aspects of the physical environment, including room size, containment (Pearson and Wilson, 2012), and seating arrangement (Rickard et al., 2020), in enhancing treatment outcomes; however, research in this area is predominantly limited to qualitative studies. Additionally, interpretation of this association is complicated by suboptimal inter-rater reliability, and the heterogenous nature of the *Therapeutic Space* variable which encompassed factors including not only the physical environment, but the appropriateness of materials, whether the task was completed with all significant family members together, and whether the family were invited to keep the drawing upon conclusion of the task after a copy was made for the clinical file.

### Limitations

While the current project provided meaningful and promising insights into the use of discourse-based coding in clinical practice, and the implementation of the *Bumps in the Road* task in family therapy, several methodological and practical limitations must be acknowledged. The current study was conducted as a pilot study within a clinical service in which the aim was much a description of “work in progress” and the research questions were exploratory. There was also the absence of a control group and small sample size. The most evident limitation was the small sample size which not only limited the power of the analysis to detect statistically significant results as previously discussed, but the scope of the statistical analyses were also limited to bivariate correlations. Although this highlighted possible associations between variables of interest for further exploration, the lack of experimental design and control group meant the relational direction between variables could not be tested, causation could not be implied, and non-significant associations should be interpreted with caution.

Further, the current study did examine the inter-rater reliability of the scale which is an important step of developing clinician-rated scales (DeVellis, 2017), however several scales require further refinement through clarification of the scale descriptions to enhance agreement amongst coders. Additionally, child mental health was the only outcome variable explored, though it would have been clinically interesting to also explore factors such as family functioning and caregiver mental health.

### Conclusions and Recommendations for Future Research

The current study was the first to present and empirically explore the *Bumps in the Road* task and associated coding instrument, which can be utilised as both a therapeutic and assessment tool. *Bumps in the Road* is also a task which has the potential to enhance coherence of attachment-related discourse, and identify maladaptive defensive processes and unresolved traumas and losses which need to be integrated into the family narrative throughout therapy. Findings discussed highlight areas for refinement in the developed coding instrument, particularly the conceptualisation and operationalisation of the attachment-family system, and the training of coders. Additionally, while the time taken to train coders (approximately 6 h in total) was significantly shorter than other discourse-based measures such as the AAI, the excessive time required to code each video (approximately 2 h) highlighted a need to work toward developing more parsimonious scales which clinicians can apply to any future transcripts or video footage.

Results also highlighted important areas for further refinement regarding the treatment manual, in particular, given findings of the importance of the therapist’s delivery of the metaphor in *Bumps in the Road*, a standardised script or further training in the theory underpinning the task may prove beneficial. Furthermore, the current study presents promising results and highlights important methodological considerations, warranting replication in a larger sample or the implementation of a dismantling study exploring whether *Bumps in the Road* itself is a mediator of treatment response in BEST-Foundations (Kazdin, 2007). While further work is still needed in integrating attachment principles into therapy for whole families, the current study provides theoretical and empirical support for the use of discourse-based coding and narrative tasks in clinical practice, to inform both formulation and intervention at a systemic level.

## Data Availability Statement

The datasets presented in this article are not readily available because the raw data are protected by Murdoch University Ethics approval (Project Reference Number: 2020/163) and cannot be made available to the general public. Requests to access the datasets should be directed to AL, Andrew.Lewis@murdoch.edu.au.

## Ethics Statement

The studies involving human participants were reviewed and approved by Human Research Ethics Committee Murdoch University. Written informed consent to participate in this study was provided by the participants’ legal guardian/next of kin.

## Author Contributions

GW performed the current research project in collaboration with and under the supervision of AL. IS was predominantly involved in collecting a large proportion of the data analysed in the current project as part of a BEST trial, and the development of a similar coding measure referred to in the current project. All authors contributed to the article and approved the submitted version.

## Conflict of Interest

The authors declare that the research was conducted in the absence of any commercial or financial relationships that could be construed as a potential conflict of interest.
