# Supplementary material for: “Bumps in the Road”: A Pilot Study of a Therapeutic Technique for the Integration of Unresolved Family Loss and Trauma
Source: Front Psychol. 2021 Jun 11;12:635574. doi: 10.3389/fpsyg.2021.635574 (PMC8226100; doi:10.3389/fpsyg.2021.635574)
Supplement: Supplementary file 1 [file Data_Sheet_1.pdf]

Supplementary Material

'Bumps in the Road' Coding Instrument

1. Therapist Intervention: Presentation of the metaphor

It is important that the therapist skilfully presents the metaphor and in doing so highlights that the story should, at minimum, include the key features of: bumps (representing traumas, losses, challenges), a car (representing the family unit), and a road (representing passage of time). In this task, there are two primary functions of the use of metaphor: (1) metaphor is used to orientate the discussion in a manner that promotes the telling of a narrative, and (2) metaphor helps to externalise problems and therefore provides a medium through which painful experiences can be discussed in a contained way.

The therapist should additionally invite the family to view the task as a creative one, allowing the family to incorporate greater nuance in their use of the metaphor, to illustrate complex situations.

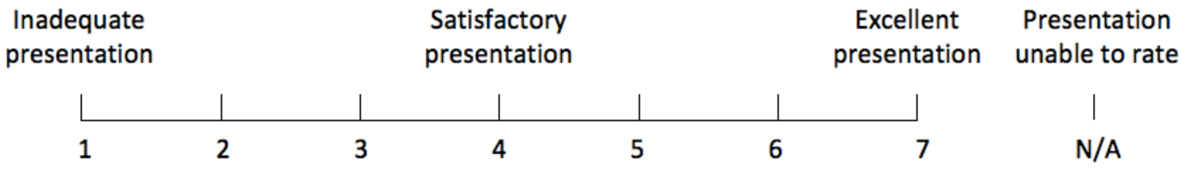

Inadequate Presentation

Therapist provides a poor presentation of the 'Bumps in the Road' metaphor, which lacks one of more key features (bumps, the car, the road), or has minimal elaboration. This may include: therapist references the presence or absence of 'bumps' with a lack of reference to magnitude or frequency; therapist explains that the 'road' represents the family journey but does not elaborate on varying paths that may be taken. It is evident that the family has misunderstood what the metaphor represents.

Satisfactory Presentation

Therapist adequately describes the 'Bumps in the Road' metaphor. This includes reference to 'bumps' of differing magnitudes ('big bumps' vs. 'small bumps') and differing frequencies (e.g., clusters of bumps, freeways). Therapist may explain that bumps can also capture experiences that were positive but challenging and/or required adjustment for the family. Therapist conveys that the 'car' represents the family unit and that the 'road' represents the family journey, and highlights varying paths that may be taken (e.g., the car may break down and need repairs, family members may get out of the car). Therapist however provides concrete examples (e.g., explicitly stating that 'family members getting out of the car' represents loss or separation) which may have a counterproductive effect on the use of the metaphor.

Excellent Presentation

Therapist clearly and succinctly describes the 'Bumps in the Road' metaphor as a creative family task and provides greater nuance in the metaphor presented. This includes: a broad variation in the use of metaphor to illustrate differing magnitudes and frequencies of events (e.g., bumps, potholes, craters, freeways); therapist explains that bumps can also capture experiences that were positive but challenging and/or required adjustment for the family and provides relevant examples, and allows permission to talk about more challenging aspects; therapist conveys that the 'car' represents the family unit and that the 'road'

represents the family journey, and highlights varying paths that may be taken, and presents this in metaphoric form (e.g., “the car can break down and need repairs but can always be fixed”, “family members may get out of the car”); therapist is able to convey the implied meaning of the task as canvassing and integrating different perspectives of challenging family events; therapist finds opportunities to refer back to the metaphor throughout the task.

**Rating:**	

**Evidence:**

Comments:

____
____
____

2. Therapist Intervention: Explanation of the activity and orientation to its therapeutic value

Providing an adequate explanation and rationale for the activity is likely to increase engagement, and will help discern whether incoherent discourse is more of a reflection of attachment-related defenses rather than a general lack of understanding around the requirements of the task. It is imperative that the therapist conveys that the goal is to co-construct a narrative or story around the family’s journey which will require elaboration and reflection, rather than simply naming the occurrence of events. As much as possible, this explanation should not bias the information later provided by family members.

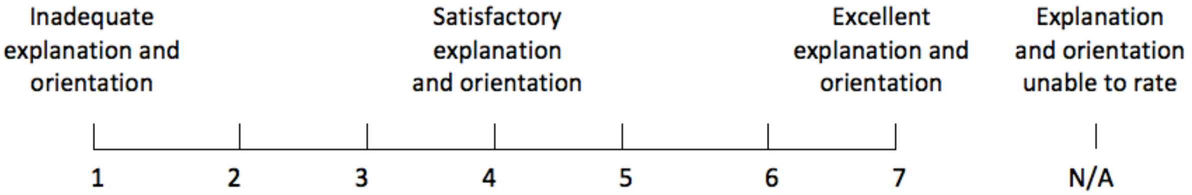

Inadequate explanation and orientation

Therapist provides an incomplete explanation of the activity and merely states or infers that ‘bumps’ are just to be identified or named. Therapist does not draw an example or illustrate how the task can be undertaken in a therapeutic manner; if an example is drawn, then it involves leading comments or inferences around family members’ roles or reactions. Therapist does not explain that the journey commences when the parents first met or first formed a relationship. Therapist in general provides an inadequate orientation of clients to the task.

Satisfactory explanation and orientation

Therapist explains that the purpose of the activity is to identify and discuss ‘bumps’, but does not highlight the importance of speaking together thoughtfully on the ‘bumps’ in terms of the family’s experience and related affect. Therapist draws a neutral example and does not make assumptions about the family. Therapist explains that the journey commences when the parents met or first formed a relationship. Therapist encourages each family member to contribute and does not guide certain family members to start. The explanation provided may be over-elaborative.

Excellent explanation and orientation

Therapist does a highly competent job of explaining that the purpose of the activity is to identify, reflect on, and discuss the experience of ‘bumps’, including mention of an affective component. Therapist draws a neutral example and does not make assumptions about the family. Therapist explains that the journey commences when the parents met or first formed a relationship. Therapist normalises the presence of ‘bumps’ in all families. Therapist encourages each family member to contribute and does not guide certain family members to start. Therapist checks for the family’s understanding of the task before proceeding. Therapist provides a succinct yet complete explanation.

**Rating:**	

**Evidence:**

Comments:

____

____

____

3. Therapist Intervention: Engagement with family discourse

The strongest indicator of attachment security on the AAI is coherence of discourse[1]. The overall aim of the ‘Bumps in the Road’ task is therefore to facilitate the development of a coherent narrative around adverse experiences; how the therapist engages with the family discourse is likely to influence whether the family discourse moves towards greater or lesser coherence. The therapist should first sit back and listen, allowing space for the story to naturally unfold, before intervening to enhance discourse structure.

[1] Main, M., Goldwyn, R., & Hesse, E. (2003). *Adult attachment scoring and classification systems.* Unpublished manuscript, University of California at Berkley.

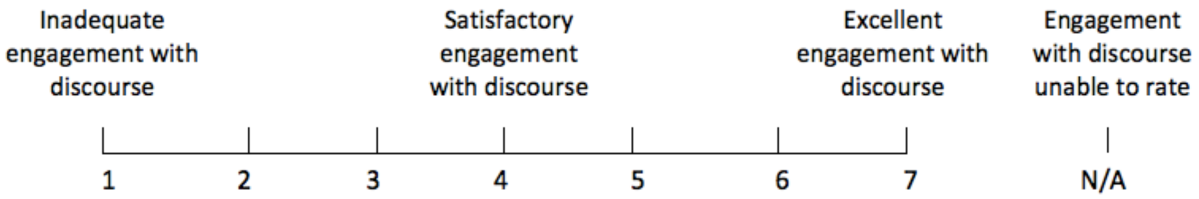

Inadequate engagement with discourse

Minimal or no clarification of confusing discourse, or inconsistent and/or implausible accounts of events or relationships. Minimal or no probing for elaboration of events. Therapist directs their attention to most dominant family member and doesn't engage the full family system. Therapist often fails to bring discourse back 'on track' when the speaker starts to provide irrelevant responses or glosses over relevant detail.

Satisfactory engagement with discourse

Therapist clarifies confusing discourse, or inconsistent and/or implausible accounts of significant events or relationships. Therapist probes for the elaboration of details and affective response to significant events. Therapist offers their own reflective insights. Therapist often encourages input from each family member. Therapist is attuned to the speaker's (conscious or unconscious) attempts to avoid the interview topic, and is able to skilfully re-direct the speaker back to the task. Therapist is able to contain family members' discourse that appears to be preoccupying (e.g., characterised by active anger or anxiety, passivity, or over-elaboration). Therapist predominantly uses open-ended questions, thus encouraging elaboration and minimising bias in the response.

Excellent engagement with discourse

Therapist almost always finds the key moments to clarify conflicting or inconsistent accounts of events or relationships. Therapist almost always probes for the elaboration of details and affective response to an event, and coping methods. Therapist encourages reflection on the impact of or meaning derived from the event. Therapist draws parallels between patterns of defensive relating across generations. Therapist consistently encourages each family member to contribute throughout the task. Therapist is attuned to the speaker's (conscious or unconscious) attempts to avoid the aims of the task, and is able to skilfully re-direct the speaker back to the task. Therapist is able to contain family members' discourse that appears to be preoccupying (e.g., characterised by active anger or anxiety, passivity, or over-elaboration). Therapist predominantly uses open-ended questions, thus encouraging elaboration and minimising bias in the response.

**Rating:**	

**Evidence:**

Comments:

---

4. Therapist Intervention: Regulation of affect

Improving affect regulation is a core component of any psychotherapeutic approach. Co-regulation of affect refers to the helping of another to modulate their emotional arousal, and enhance understanding and containment of affective experiences[2]. This occurs through attunement to another's affect as conveyed through their nonverbal cues (e.g., bodily expressions, voice prosody), and responding, verbally or nonverbally, in a congruent, warm, and empathetic manner. This process of co-regulation creates a sense of safety and allows the exploration and reflection of distressing events.

During this task, the therapist should facilitate affective regulation of the family system as needed, modelling effective co-regulation skills, and where appropriate, encouraging caregivers to co-regulate their child's affective experiences.

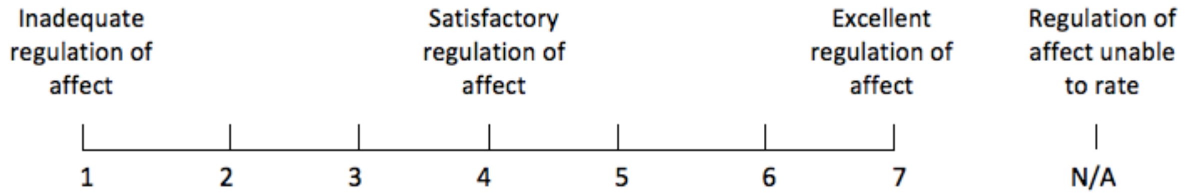

Inadequate regulation of affect

Therapist displays poor affective regulation of the attachment-family system during the task. Therapist fails to highlight affective valence of significant events or fails to address conflict, anger, or aggression shown in the account given. Therapist is misattuned to the moment-by-moment affective experience of the family, which may lead to escalations in distress or withdrawal. Therapist provides responses to emotional expression which may be considered dismissive, invalidating, or insensitive, or may become dysregulated themselves.

Satisfactory regulation of affect

Therapist displays adequate affective regulation of the attachment-family system during the task. Therapist encourages the expression of affective experience relating to significant events and addresses conflict, anger, or aggression shown in the account given. Therapist responds to emotional expression through matching the affect through their verbal and nonverbal behaviour (e.g., facial expressions, gestures, voice prosody), thus communicating their understanding, all while remaining regulated themselves. Therapist remains attuned to the affect of the family system at most times, and interacts with the family in a warm, validating, and empathetic manner. Therapist co-regulates the affect of family members consequently modelling appropriate skills, but in doing so does not adopt a systemic approach.

[2] Hughes, D. (2007). *Attachment-focused family therapy*. W.W.Norton.

Excellent regulation of affect

Therapist is highly competent in facilitating the affective regulation of the attachment-family system during the task. Therapist facilitates the expression and understanding of affective experiences relating to significant events, and addresses conflict, anger, or aggression shown in the account given. Therapist responds to emotional expression through matching the affect through their verbal and nonverbal behaviour (e.g., facial expressions, gestures, voice prosody), thus communicating their understanding, and while remaining regulated themselves. The therapist remains attuned to the affect of the family system at any given time (e.g., addresses avoidance, dysregulation, or withdrawal), and interacts with the family in a warm, validating, and empathetic manner. Therapist displays a high level of emotion awareness and finds key opportunities to encourage and coach caregivers to co-regulate their child's or partner's distress in-the-moment.

**Rating:**	

**Evidence:**

Comments:

____
____
____

5. Therapist Intervention: Therapeutic space and materials

Consideration should be given to the set-up of the therapy room within which this task occurs, given research indicating that aspects of the physical environment are associated with the therapy process itself[3]*. This task minimally requires the use of a therapeutic space that is appropriate, contained, and promotes the family working together with guidance from the therapist/s only when necessary. The physical environment (including seating arrangements and access to required materials) should not constrain engagement in the task.*

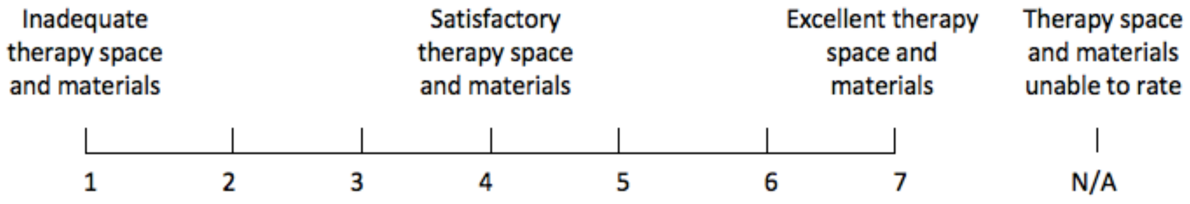

[3] Pearson, M. & Wilson, H. (2012). Soothing spaces and healing places: Is there an ideal counselling room design? *Psychotherapy in Australia, 18*(3), 46-53. https://www.researchgate.net/publication/254724357_Soothing_spaces_and_healing_spaces_Is_there_an_ideal_counselling_room

Inadequate therapy space and materials

Task was completed with parents and children in separate rooms. Physical environment was inappropriate, e.g., small, noisy, cluttered, open. Seating arrangement was inappropriate, e.g. confrontational or intrusive. Required supplies were insufficient, e.g. small piece of paper, shared pen, small table. Therapist does not retain a copy of the drawing or provide this to the family.

Satisfactory therapy space and materials

Task was completed with parents and children together. Physical environment was contained and appropriate, e.g., quiet therapy room, clear of clutter. Seating arrangement was non-confrontational and non-intrusive, however the therapist was seated with the family, and/or some members may have had difficulty reaching the table and/or pens to freely participate in the task. Therapist invites the family to keep the final drawing upon conclusion of the task.

Excellent therapy space and materials

Task was completed with parents and children together. Physical environment was contained and appropriate, e.g., quiet therapy room, clear of clutter. Seating arrangement was well considered, so as to promote collaboration among the family system. This included: the therapist/s were seated in a non-confrontational and non-intrusive manner or 'hovered' around the family during completion of the task; a sufficiently sized table was positioned in the centre of the room with family members circulating the table, each able to reach the required materials. Family members were offered a choice in their seating, as this may provide valuable clinical information. Required supplies and space were sufficient for the number of people involved. Therapist takes a photograph or copy of the final drawing for the clinical file, and invites the family to keep the final drawing upon conclusion of the task to continue to develop collaboratively outside of therapy.

**Rating:**	

**Evidence:**

Comments:

____

____

____

1. Family Discourse: Coherence

The strongest indicator of attachment security on the AAI is coherence of discourse[4]. Discourse coherence in the current context refers to the presentation of an overall narrative that is convincing (e.g., both internally consistent and externally plausible), balanced (e.g., not overly positive or negative), evidences reflection, and in which the family system engages in collaborative conversation with each other and with the therapist, to fulfil the requirements of the task.

Violations of Grice's maxims[5] are taken into consideration when coding for coherence, these are: quality (e.g., claims are supported with evidence, minimal contradictions), quantity (e.g., succinct and complete depictions of experiences), relation (e.g., information provided is relevant to the agreed task, and manner (e.g., discourse is clear and understandable to the listener).

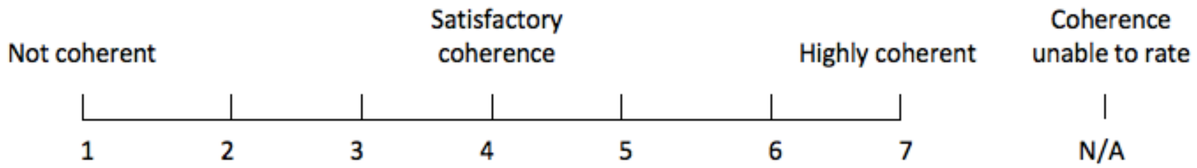

Not Coherent

Family discourse indicates significantly contradictory recollections of experiences, relationships, and their impacts. Family members are non-cooperative with the task, and do not engage in collaborative exchanges with the therapist or each other. Family members gloss over, avoid, or minimise significant traumas (that were experienced either directly by the family, or that were experienced by the parents but may continue to significantly influence caregiving behaviour), thereby failing to integrate them into the family narrative and undermining overall coherence. Listener/therapist has significant difficulty comprehending most speech due to consistent violations of Grice's maxims (e.g., contradictions, irrelevant information, vague speech, use of jargon). Family members who have had some involvement in the therapy, were not present for the task despite being invited (indicating an absence or failing to contribute to the family narrative).

Satisfactory coherence

Family members engage well with the task and remain cooperative and collaborative with each other and the therapist for most of the interview. This is balanced with clear violations of Grice's maxims, or non-cooperative or non-collaborative discourse around a few events or experiences (with the exception of significant losses or traumas). Family members raise almost all significant traumas and are largely able to discuss these with few violations of Grice's maxims. One parent may offer little contribution to the narrative, however this is balanced with another parent who shows evidence of coherence in the narrative they provide. Overall, the listener/therapist has a relatively good understanding of the narrative provided around adverse experiences.

[4] Main, M., Goldwyn, R., & Hesse, E. (2003). *Adult attachment scoring and classification systems.* Unpublished manuscript, University of California at Berkley.

[5] Grice, H. P. (1975). Logic and conversation. In P. Cole & J. L. Moran (Eds.), *Syntax and semantics: Vol. 3. Speech Acts* (pp. 41-58). New York: Academic Press; Grice, H. P. (1989). *Studies in the way of words.* Cambridge: Harvard University Press.

Highly coherent

Family members display a single, consistent narrative around experiences and their impact on family members. Family members are able to engage well with the task, collaborate together, and contribute appropriately. Parents are able to speak about past traumas in a child-focused way and can remain regulated enough themselves to respond sensitively to the child/ren's needs throughout the task. Family members raise all significant traumas (from the start of the 'road' to the present) and are able to speak about these with minimal violations of Grice's maxims. Speaker engages in "licensing" if they proceed to violate a maxim (e.g., "this might be a long story…"). Family discourse evidences 'meta-cognitive monitoring' e.g., self-correcting any inconsistencies within the discourse based on contributions or recognition of these inconsistencies. Family members develop new insights as the task progresses. Overall, the listener/therapist is well persuaded around the plausibility of the narrative and has no difficulty understanding the discourse.

**Rating:**	

**Evidence:**

Comments:

2. Family Discourse: Deactivation

Deactivation is an attachment-related defense characterised by the dismissal or devaluation of attachment-related experiences[6].

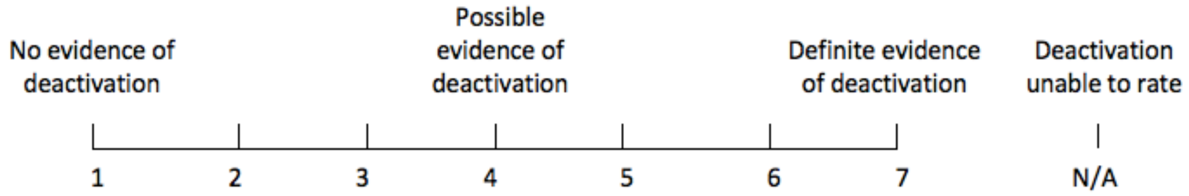

Indicators:

1. Provides overly favourable and unsupported descriptions of events:
   (a) Minimises the difficulty or severity of events.
   (b) Unwillingness, inability, or significant difficulty identifying bumps (includes insistence on lack of memory in a manner that blocks further discourse).

[6] Bowlby, J. (1980). *Attachment and loss: Loss* (Vol. 3). New York, NY: Basic Books.

(c) Provides overly succinct explanations of experiences and are unwilling or unable to sufficiently elaborate when prompted.
(d) Difficult experiences are normalised, or generic responses are provided.
(e) Narrative around experiences conveys displacement or denial of distress as a means of coping.

2. Attachment relationships or experiences are devalued, dismissed, or derogated:
   (a) Family discourse during the task reflects dismissal or devaluation of another's attachment needs (including lack of sensitivity, and/or caregiver's disproportionate focus on child's "problem behaviours" with limited acknowledgement of own challenges).
   (b) Family discourse emphasises discipline over emotional connectedness and warmth.

3. Emphasis on non-attachment-related or non-affective aspects of relationships or experiences:
   (a) Narrative around difficult events is not personalised (e.g., "There was a miscarriage" rather than "We/I had a miscarriage")
   (b) Discourse conveys non-interpersonal strategies for assuagement, e.g. just 'getting over it', 'not thinking about it', immersing oneself in work.
   (c) Narrative overemphasises practical aspects of relationships or is highly detail-oriented, at the expense of conveying the subjective, affective experience.

**Rating:**	

**Evidence:**

Comments:

____

____

____

3. Family Discourse: Disconnection

Disconnection is an attachment-related defense characterised by affective or behavioural expression becoming disconnected from the attachment-related source[7].

[7] Bowlby, J. (1980). *Attachment and loss: Loss* (Vol. 3). New York, NY: Basic Books.

1	2	3	4	5	6	7	N/A
No evidence of disconnection			Possible evidence of disconnection			Definite evidence of disconnection	Disconnection unable to rate

Indicators:

1. Attachment experiences are pre-occupying:
   (a) Over-elaboration when describing an event or experience.
   (b) Narrative repeatedly references the same preoccupying events or attachment relationships.
   (c) Fixation on irrelevant details of the event, correcting and clarifying, rather than moving on to tell the overall story.

2. Indicators of emotional or behavioural reactions that are disconnected from their attachment-related source:
   (a) Evidence of behaviours that keep the other close are presented in the narrative or displayed during the task (including displays of clinging behaviour to gain the caregiver's attention).
   (b) Heightened emotion is evident in the discourse (anger, anxiety).
   (c) Theme of narrative oscillates between a desire for closeness and withdrawal.
   (d) Fear of loss of the child through death (note: do not score if source of fear is conscious and fear is not impacting on behaviour).
   (e) Discourse indicates psychological merging between family members.
   (f) One caregiver controlling the narrative with limited integration of others' experiences.

3. Discourse is vague or confusing:
   (a) Vague speech e.g. use of "sort of thing", "or something" (note: consider strength and frequency, and whether this violates overarching maxim of collaborative conversation, and consider whether narrative is vague because the event has been discussed in detail in previous sessions).
   (b) Uncertainty or confusion regarding people or events.

**Rating:**	

**Evidence:**

Comments:

4. Family Discourse: Segregated Systems (Trauma)

Segregated Systems is an attachment-related defense characterised by multiple, unintegrated mental models of attachment[8]. ***A rating of 4 and above on this scale should be taken into consideration when coding for overall coherence of discourse.***

Here, code only evidence of segregated systems during specific discussions of trauma, whereby trauma can comprise events including perceived threats to life, significant illnesses, and displays of frightening behaviour by caregivers (e.g., abuse, parental mental health, exposure to family and domestic violence).

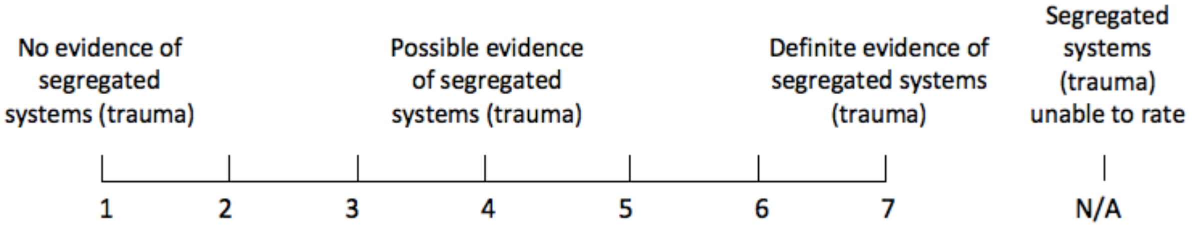

Indicators during specific discussions of traumatic events:

1. Discourse is odd, unclear, or disorganised:
   (a) Odd, unexplained comments.
   (b) Prolonged silences.
   (c) Significant contradictions in key details regarding traumatic events provided by family members.
   (d) Unusual attention to detail when describing the event.
   (e) Evidence of intrusion from potentially dissociated belief systems, e.g. disorientation with respect to space and time, poetic phrasing, intrusions of information related to the abuse into other topics, odd associations present in discourse.[9]

2. Emotional/behavioural dysregulation or constriction is evident in the family discourse:
   (a) Verbal or physical withdrawal from discussions around traumas with no acknowledgement of this by the family (e.g., "I'd rather not speak about that").
   (b) Flow of discourse is abruptly altered to prevent breakdown.
   (c) Unnatural shifts of conversation away from the topic of loss.
   (d) Displays of incongruent affect.
   (e) Displays of conflicting attachment behaviours in the family discourse, e.g., verbally comforting but physically moving away.
   (f) Evidence of frightening or frightened caregiving.
   (g) Discourse conveys caregiver lack of resources to cope leading to withdrawal
   (h) Developmentally-inappropriate parentification of children or role reversal

3. Narrative perpetuates the lack of integration and resolution of the events:

[8] Bowlby, J. (1980). *Attachment and loss: Loss* (Vol. 3). New York, NY: Basic Books.

[9] *Note: this only needs to be present in the discourse of one family member to be able to be coded, as displays of frightening caregiving behaviour due to the operation segregated systems, are theorised to contribute to the development of disorganised attachment in children.*

(a) Statements which imply (and perhaps therefore normalise), psychological attempts to dissociate or divide the mind as a means of coping with the trauma.
(b) Narrative is characterised by themes of helplessness, fear, or failed protection.
(c) Absence of any form of discourse around a traumatic event that was directly experienced by the family.
(d) Unusual beliefs regarding the abuse, such as denial, dismissal, and inappropriate self-blame.
(e) Evidence of detachment from trauma narrative.

**Rating:**	

**Evidence:**

Comments:

5. Family Discourse: Segregated Systems (Loss)

*Segregated Systems is an attachment-related defense characterised by multiple, unintegrated mental models of attachment[10]. **A rating of 4 and above on this scale should be taken into consideration when coding for overall coherence of discourse.***<br><br>*Here, code only evidence of segregation during specific discussions of loss, including pregnancy losses and loss of pets. Do not code if indicators of segregation are only present within losses occurring within the preceding year.*

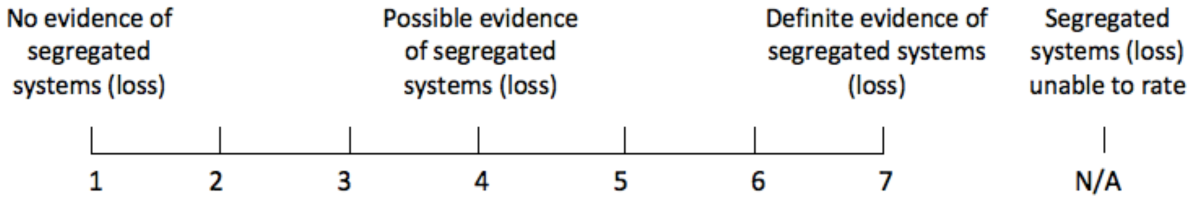

Indicators during specific discussions of loss experiences:

1. Narrative is odd, unclear, or disorganised:
   (a) Odd, unexplained comments.
   (b) Prolonged silences.

[10] Bowlby, J. (1980). *Attachment and loss: Loss* (Vol. 3). New York, NY: Basic Books.

(c) Significant contradictions in key details regarding loss experiences provided by family members.
(d) Unusual attention to detail when describing the event.
(e) Evidence of intrusion from potentially dissociated belief systems, e.g. disbelief that a person is dead, confusion between dead person and self, disorientation with respect to space and time, poetic phrasing, intrusions of information related to the loss into other topics, odd associations present in discourse.

2. Emotional/behavioural dysregulation or constriction is evident in the family discourse:
   (a) Verbal or physical withdrawal from discussions around loss with no acknowledgement of this by the family (e.g., "I'd rather not speak about that").
   (b) Flow of discourse is abruptly altered to prevent breakdown.
   (c) Unnatural shifts of conversation away from the topic of loss.
   (d) Displays of incongruent affect.
   (e) Displays of conflicting attachment behaviours, e.g., verbally comforting but physically moving away.

3. Narrative perpetuates the lack of integration and resolution of the loss:
   (a) Statements which imply (and perhaps therefore normalise) psychological attempts to dissociate or divide the mind as a means of coping with distress.
   (b) Absence of any form of discourse around a significant loss that was directly experienced by the family.
   (c) Unusual beliefs regarding the loss, such as denial, dismissal, and inappropriate self-blame.
   (d) Evidence of detachment from loss narrative.

**Rating:**	

**Evidence:**

Comments:
____
